# Towards a Clinical Decision Support System for External Beam Radiation Oncology Prostate Cancer Patients: Proton vs. Photon Radiotherapy? A Radiobiological Study of Robustness and Stability

**DOI:** 10.3390/cancers10020055

**Published:** 2018-02-18

**Authors:** Seán Walsh, Erik Roelofs, Peter Kuess, Yvonka van Wijk, Ben Vanneste, Andre Dekker, Philippe Lambin, Bleddyn Jones, Dietmar Georg, Frank Verhaegen

**Affiliations:** 1 Department of Radiation Oncology (MAASTRO), GROW—School for Oncology and Developmental Biology, Maastricht University Medical Centre, Doctor Tanslaan 12, Maastricht 6229 ET, The Netherlands; walshsharp@gmail.com (S.W.); erik.roelofs@maastrichtuniversity.nl (E.R.); ben.vanneste@maastro.nl (B.V.); andre.dekker@maastro.nl (A.D.); 2 The D-Lab: Decision Support for Precision Medicine, GROW—School for Oncology and Developmental Biology, Maastricht University Medical Centre, Universiteitssingel 40, Maastricht 6229 ER, The Netherlands; y.vanwijk@maastrichtuniversity.nl (Y.v.W.); philippe.lambin@maastrichtuniversity.nl (P.L.); 3 Gray Laboratory, CRUK/MRC Oxford Oncology Institute, University of Oxford, ORCRB-Roosevelt Drive, Oxford OX3 7DQ, UK; 4 Department of Radiation Oncology and Christian Doppler Laboratory for Medical Radiation Research for Radiation Oncology, Medical University of Vienna, 1090 Vienna, Austria; peter.kuess@meduniwien.ac.at (P.K.); dietmar.georg@akhwien.at (D.G.); 5 Gray Laboratory, CRUK/MRC Oxford Oncology Institute, The University of Oxford, ORCRB-Roosevelt Drive, Oxford OX3 7DQ, UK; bleddyn.jones@oncology.ox.ac.uk

**Keywords:** prostate cancer, radiotherapy, proton therapy, clinical decision support systems, in silico trial, radiobiological modelling

## Abstract

We present a methodology which can be utilized to select proton or photon radiotherapy in prostate cancer patients. Four state-of-the-art competing treatment modalities were compared (by way of an in silico trial) for a cohort of 25 prostate cancer patients, with and without correction strategies for prostate displacements. Metrics measured from clinical image guidance systems were used. Three correction strategies were investigated; no-correction, extended-no-action-limit, and online-correction. Clinical efficacy was estimated via radiobiological models incorporating robustness (how probable a given treatment plan was delivered) and stability (the consistency between the probable best and worst delivered treatments at the 95% confidence limit). The results obtained at the cohort level enabled the determination of a threshold for likely clinical benefit at the individual level. Depending on the imaging system and correction strategy; 24%, 32% and 44% of patients were identified as suitable candidates for proton therapy. For the constraints of this study: Intensity-modulated proton therapy with online-correction was on average the most effective modality. Irrespective of the imaging system, each treatment modality is similar in terms of robustness, with and without the correction strategies. Conversely, there is substantial variation in stability between the treatment modalities, which is greatly reduced by correction strategies. This study provides a ‘proof-of-concept’ methodology to enable the prospective identification of individual patients that will most likely (above a certain threshold) benefit from proton therapy.

## 1. Introduction

Prostate cancer is the most common cancer diagnosis and the third-leading cause of cancer death [1]. When prostate cancer is suspected, biopsy is the standard of care for diagnosis. Though, the emergence of advanced imaging and biomarkers [2] has improved risk stratification through precise identification and characterization of the disease. Several treatment options are available for prostate cancer patients. For metastatic disease, chemotherapy as initial treatment now appears to extend survival compared with hormone therapy alone. For localized disease, active surveillance appears to be safe and has become the preferred approach for low-risk patients, surgery and radiotherapy continue to be curative treatments for intermediate/high-risk patients but have adverse effects that can negatively affect quality of life. In the context of radiotherapy treatment, there are several options: brachytherapy and external beam radiotherapy (photons, protons and ions). With respect to a definitive assessment of the clinical efficacy of proton (P-EBRT) vs. photon (X-EBRT) external beam radiotherapy cannot be made as there is limited evidence [3]. Therefore, clinicians are faced with a dilemma when deciding which treatment option to utilize. This should be viewed against the backdrop of the move towards precision medicine [4,5] (the right treatment for the right patient) within the healthcare community.

In silico trials offer a potential solution to this dilemma as they allow multiple-simulation of virtual randomized clinical trials for different treatment modalities with different treatment strategies for the same patients, facilitating direct like-for-like quantitative comparisons of probable clinical outcomes via radiobiological models [6,7,8]. ROCOCO (Radiation Oncology Collaborative Comparison) [9,10,11,12,13] is a multicentric in silico trial which compares X-EBRT and P-EBRT, in this instance for 25 high-risk prostate cancer patients. In silico trials have been performed in both lung [14] and head-and-neck cancer [15] and are a recognized model-based approach for the realization of precision medicine [16]. This in silico trial is designed to accurately reflect clinical reality by incorporating factors such as heterogeneity in anatomy, radiosensitivity, and target motion. Image guidance systems such as three-dimensional ultrasound (3DUS) [17] and cone-beam computed tomography (CBCT) [18] provide target localization before and during treatment. These systems identify and correct problems arising from inter- and intrafractional variations in patient setup and anatomy. The treatment techniques compared are intensity-modulated radiotherapy (IMRT—varying the photon energy fluence, and subsequent dose, across a radiation therapy treatment field by intersecting the nonuniform dose distributions from multiple treatment fields enabling a high degree of dose conformity around the intended target and increased normal structure sparing [19]), volumetric-modulated arc therapy (VMAT—delivers IMRT treatment in a continuous single- or double-arc gantry rotation [20]), passively scattered proton therapy (PSPT—a form of radiation treatment that uses high-energy proton beams to irradiate tumors, the principal feature and physical advantage of proton therapy is the finite range of protons, delivering a reduced dose proximal to the target volume and essentially no dose beyond the end of their range [21]), and intensity-modulated proton therapy (IMPT—A technique that allows for three-dimensional dose conformity to a target volume using protons through pencil-beam scanning with dynamic control and optimization of the beam energy and intensity throughout the scan [22]).

Notwithstanding the many positive attributes of trials, there is an increasing belief that equating all evidence-based medicine with trials is an undue simplification [16] and as a consequence, randomizing (non-enriched) cohorts between X-EBRT and P-EBRT is predictably inefficient and likely to produce confusing results. Here we explore this contention by performing an in silico trial and reporting the results at both the cohort and patient levels, enabling the direct comparison of the conventional method (non-enriched populations) to produce evidence [23] and a proposed alternative method known as the model-based approach (enriched populations).

This study, within the context of prostate cancer patients, addresses technical issues related to delivering scientific evidence for the application of precision radiation oncology (e.g., X-EBRT vs. P-EBRT for any given patient), in effect providing an innovative methodology for utilization in a clinical decision support system (CDSS) for prostate cancer patients.

## 2. Materials and Methods

### 2.1. Treatment Modalities

**Figure 1:**
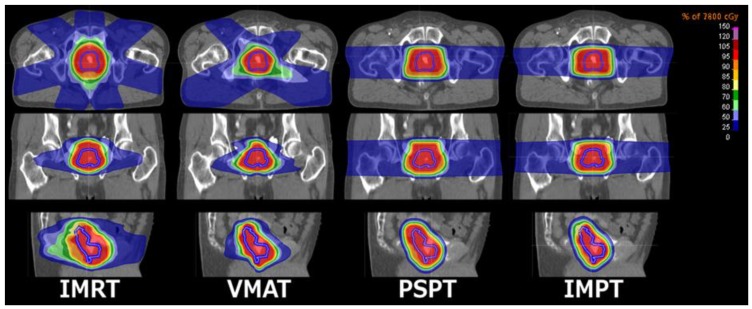
Dose distribution for each treatment modality: displayed in the transverse, coronal and sagittal planes. The clinical target volume is contoured in blue.

We considered four state-of-the-art competing X-EBRT and P-EBRT techniques. The planned dose distributions for an example patient for each modality are depicted in Figure 1.

(1) **IMRT** plans were generated at MAASTRO Clinic, the Netherlands, through seven treatment fields (0°, 53°, 104°, 154°, 206°, 256°, 307°) optimized on the planning target volume (PTV) with leaf positions and 6MV photons, planned with RayStation (RaySearch, Stockholm, Sweden).
(2) **VMAT** plans were generated at MAASTRO Clinic, the Netherlands, through dual anterior arcs (1°–359°, 359°–1°) with 91 control points per arc, optimized on the PTV with leaf positions and 6MV photons, planned with RayStation.
(3) **PSPT** plans were generated at UPenn RPTC, USA, through two treatment fields (90°–270°) optimized on the clinical target volume (CTV) with individualized beam apertures, range compensators, and an assumed constant relative biological effectiveness (RBE) of 1.1. Dose smearing and aperture expansion of 10 mm as well as 3.5% range uncertainty distally and proximally were allowed for, planned with XiO (Elekta).
(4) **IMPT** plans were generated by MAASTRO Clinic, the Netherlands, using two treatment fields (90°–270°) optimized on the CTV through pencil beam scanning and an assumed constant RBE of 1.1. Range uncertainty margins of 3.0% + 1.5 mm were allowed for distally and proximally, planned with RayStation.

### 2.2. Treatment Planning

Treatment planning (TP) was based upon international commission on radiation units and measurements (ICRU) dose prescription criteria [24,25]. Exact dose volume histogram (DVH) planning criteria are tabulated in Table 1. The PTV was a 4 mm isotropic expansion of the CTV. No hot spots were allowed outside of the PTV. The dose was to be delivered at 2 Gray (Gy) or isoeffective Gray Equivalent (GyE) per fraction.

Organ at risk (OAR) sparing was prioritized in the following order: rectal-wall, sigmoid-colon, bladder, small intestines, femoral heads and skin. The TP constraints used for these OARs followed the published recommendations of quantitative analyses of clinical normal tissue effects criteria (QUANTEC) [8,26]. Dose calculation was computed with tissue heterogeneity correction activated, using a superposition/convolution or collapsed cone algorithm. Because there was contrast medium present in the bladder during the computed tomography (CT) scan but not during treatment, voxels with contrast medium were forced to water equivalent electron density.

### 2.3. Conformity Index

The conformity index (COIN) [27] provides a quantitative evaluation of the degree of conformity and was calculated for the PTV for each treatment plan for each modality, please see Supplementary Material subsection ‘Conformity index’. COIN is shown in Table 1 as a reference metric.

### 2.4. The Radiobiological Models

The radiobiological models and parameter assumptions used in this study have been extensively described previously [6,7,8] and were used to evaluate each treatment modality. Concisely, the tumor control probability (TCP) model predicts 5-year biological no evidence of disease (5y-bNED) and the normal tissue complication probability (NTCP) model predicts radiation therapy oncology group (RTOG) Grade ≥2 late rectal toxicity e.g., rectal bleeding (as the rectum is the most dose-limiting structure in prostate cancer EBRT [28]).

### 2.5. Prostate Displacement Relative to Initial Skin-Mark-Laser Alignment

Displacement probability distributions were provided by 3DUS which measured inter-fraction prostate displacements for 56 patients treated at University Hospital Galway—Saolta University Health Care Group, Ireland. Published data for CBCT imaging [29] was also considered. The corresponding metrics are tabulated in Table S1 (please see supplementary material). The displacement statistics of the prostate are distilled into systematic and random displacement components [30]. The systematic component describes a constant shift between planning and treatment anatomy. The random component refers to treatment execution, reflecting day-to-day variations about a systematic displacement. The systematic errors cause a shift of the dose distribution, while the random errors will cause a blurring of the dose distribution.

### 2.6. Correction Strategies

The no-correction strategy relies entirely on the plan/PTV to ensure that the prescription dose is delivered to the CTV during treatment.

The extended-no-action-limit-(eNAL)-correction strategy [31] involves the estimation of the systematic treatment error after 3 fractions and an isocentre realignment correction performed which is the average systematic error vector. The eNAL also includes once-weekly imaging to monitor the correction; if the prostate is located within tolerance no-action is taken, if out of tolerance further images are obtained to re-determine the systematic error. It is suggested [31] that only systematic errors >2 mm be corrected. Currently the eNAL has only been implemented in X-EBRT clinical practice.

The online-correction strategy consists of daily target localization, and isocentre realignment if necessary, to ensure that the prescription dose is delivered to the CTV during treatment. Online-correction is widespread in X-EBRT clinical practice; however, it is not universally utilized in P-EBRT clinical practice, although the need is recognized [32].

### 2.7. The Simulation of Treatment

The metrics in Table S1 through a plan robustness analysis module were used to simulate the displacement of the prostate. Rigid probabilistic prostate displacements were simulated for each treatment fraction. Treatment, consisting of 39 fractions, was simulated 100 times for each patient. Dose-voxel tracking is computed during simulation, based upon displacements of the prostate and geometrical isocenter realignment if performed (If correction is performed it is assumed to be perfect, i.e., error <1 mm).

Dose recalculation is typically not required for X-EBRT treatment of the prostate [33]; conversely, dose recalculation is typically assumed to be required for all P-EBRT treatment. Therefore, the scripting functionality in RayStation [34] (http://www.raysearchlabs.com/automated-treatment-planning/#scripting) was used to simulate the displacement of the prostate for P-EBRT (implemented in IronPython: Full dose recalculation).

### 2.8. Robustness, Stability, and Score

The conventional definition of robustness is the confidence that a plan metric will be delivered within limits with a high probability, and has been fully described previously [35]. In this study we utilize a similar but alternative approach, defining robustness, stability and score as:

(1) $$\mathrm{Robustness}=1-({\mathrm{TCP}}_{\mathrm{plan}}-{\mathrm{TCP}}_{\mathrm{median}})$$

(2) $$\mathrm{Stability}=1-{\Delta \mathrm{TCP}}_{95\%\mathrm{CI}}$$

(3) $$\mathrm{Score}=\left({\mathrm{TCP}}_{\mathrm{plan}}-{\mathrm{NTCP}}_{\mathrm{plan}}\right)\times \mathrm{Robustness}\times \mathrm{Stability}$$

TCP_plan_ and NTCP_plan_ are obtained from the DVHs for the CTV and rectal-wall respectively for the planned treatment. TCP_median_ is obtained from the median DVH for the CTV, produced from the 100 simulated treatments. Robustness indicates how likely a given treatment plan was executed in terms of TCP with respect to the plan. Stability indicates the range of TCP for the simulated treatments at the 95% confidence interval double-sided. The NTCP_plan_ provides a control metric for the initial treatment plan. Score combines all these quantities into a single figure of merit ranging from −1 (zero probability of tumor control with certain rectal complication) to +1 (certain tumor control with zero probability of rectal complication). The philosophy of the score metric is based upon the following: TCP provides the probability of tumor control, while NTCP provides the probability of toxicity of normal tissue, the maximum difference between the two is known as the ideal therapeutic index. This constitutes the first aspect of the score metric. However, the probability of the therapeutic index must be placed in the context of the second aspect of the score metric, robustness and stability. Robustness provides the probability of achieving the most probable therapeutic index. Stability provides the probability of convergence between the best probable and worst probable therapeutic indices.

### 2.9. Summary of the In Silico Trial Workflow

**Figure 2:**
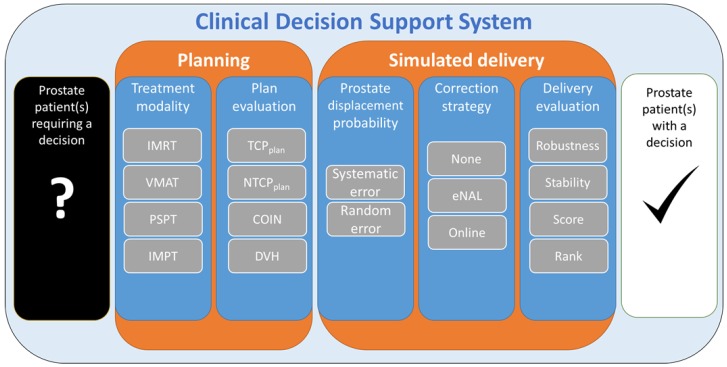
Workflow of this in silico trial (from left to right): The process begins with a prostate patient dataset. Each dataset is entered into the planning stage, where a plan is created for all possible treatment modalities. Subsequently, each plan is evaluated by dose metrics and radiobiological models. Next, each plan is entered into the simulated delivery stage, where known likely clinical errors (target motion) along with correction strategies are introduced/simulated into the plan/delivery. Subsequently, each plan is evaluated in terms of robustness and stability, which in turn produces a score and finally a rank. This enables two conclusions to be made for these clinical conditions, planning criteria, and simulations parameters: (1) which modality is ranked highest across the cohort, and (2) which modality is ranked highest for each individual patient.

The workflow for this study is depicted in Figure 2.

## 3. Results

**Figure 3:**
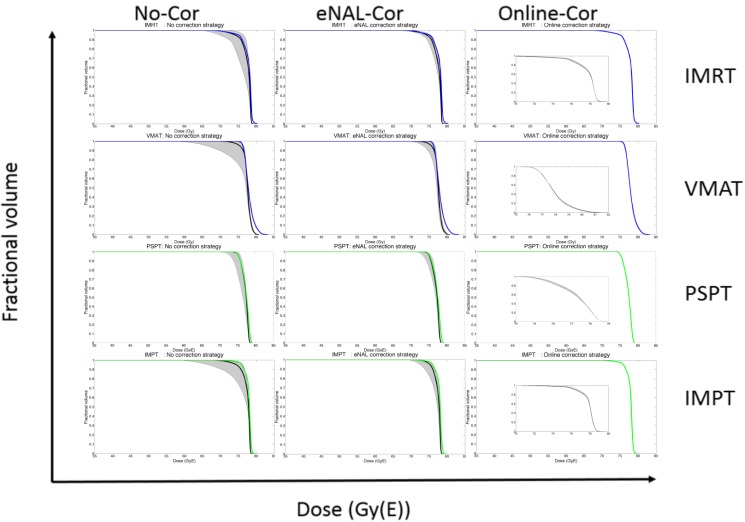
CTV-DVH data for an example patient for IMRT, VMAT, PSPT and IMPT with and without correction: The colored lines represent the planned treatment (blue X-EBRT, green P-EBRT). The solid black lines represent the median treatment delivered and is related to robustness which denotes the likelihood of delivering the planned dose. The shaded grey regions depict the 95% confidence intervals and are related to stability which denotes the possible range of the dose delivered. The distribution of possible treatments is asymmetric. The small figures inside the right column of the figure is a magnification of the dose-drop-off region of the DVH.

For all treatment modalities; satisfactory CTV and PTV coverage was achieved and OAR constraints were met for all patient plans. To illustrate the effect of inter-fraction prostate displacement on the CTV dose to be delivered, an example patient to be treated by IMRT, VMAT, PSPT and IMPT is presented in Figure 3.

Each modality was evaluated based upon prostate displacement probability distributions provided by 3DUS and CBCT, with and without correction strategies. The mean, standard deviation and range for all the assessment metrics are listed in Table 2.

At the cohort level: TCP_plan_ was highest for IMPT 56 ± 11% (range: 30–70%) and lowest for VMAT 49 ± 13% (range: 14–67%). NTCP_plan_ was lowest for IMPT 10 ± 3 (range: 5–17%) and highest for IMRT 12 ± 3% (range: 7–17%). IMRT_3DUS_ (no-correction) was least robust 97 ± 3% (range: 89–100%) and VMAT_CBCT_ (online-correction) was most robust 101 ± 0% (range: 100–103%). Improved conformity resulted in less stable treatment. The correction strategies greatly improve stability; by a maximum of 19% (eNAL-correction) to 24% (online-correction) for IMPT_CBCT_ and a minimum of 11% (eNAL-correction) to 18% (online-correction) for PSPT_CBCT_. However, the correction strategies have limited influence on robustness; a maximum improvement of 0% (eNAL-correction) to 3% (online-correction) for IMPT_CBCT_ and a minimum of 0% (eNAL-correction) to 0% (online-correction) for IMPT_3DUS_.

**Figure 4:**
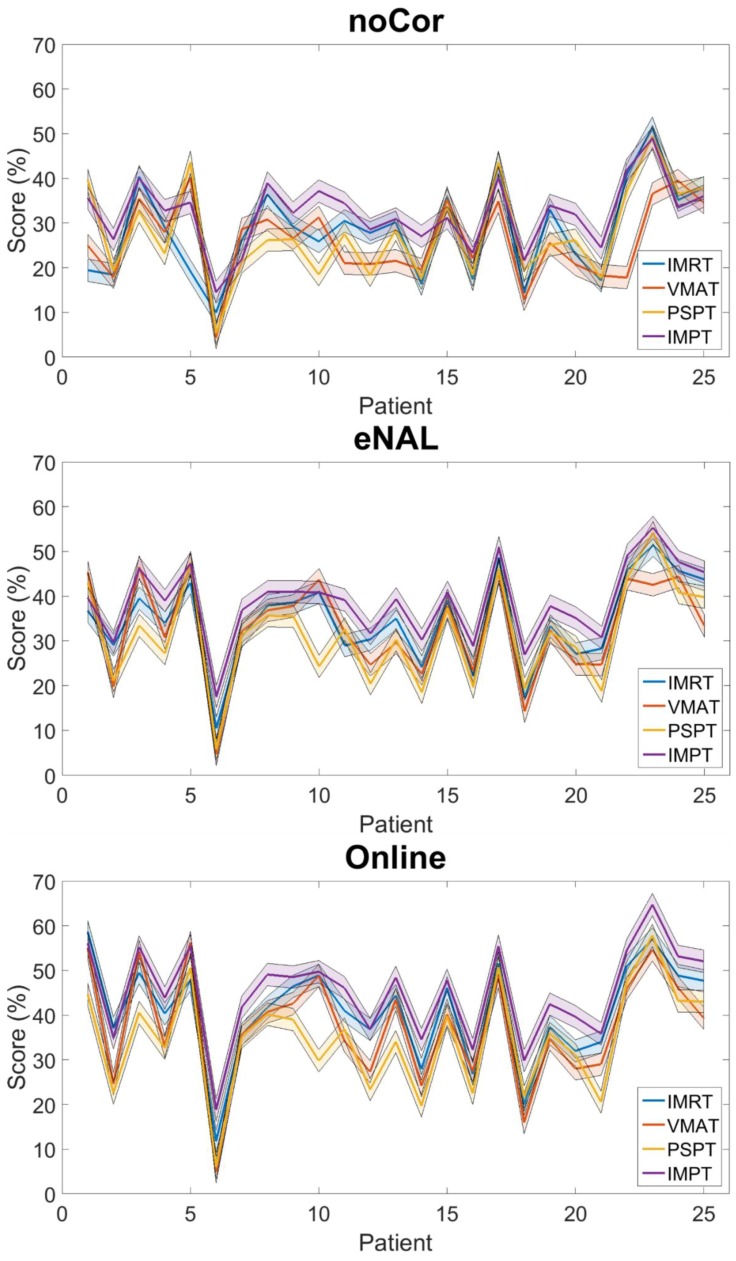
Patient stratification: The colored lines represent each treatment modality. The shaded region represents the threshold of likely clinical benefit, in this instance 5%. The correction strategies consistently improve the patient score, while each patient exhibits considerable variability per modality.

The results obtained at the cohort level reported in Table 2 enabled the determination of a threshold for likely clinical benefit, in this instance 5%. Therefore, each patient was retrospectively stratified accordingly into X-EBRT or P-EBRT. For no-, eNAL- and online-correction, 24%, 32% and 44% of patients were identified as suitable candidates for P-EBRT, respectively. The individual patient scores for each modality and correction strategy are depicted in Figure 4. Detailed analysis of patient stratification is provided in the Supplementary Materials: Tables S2–S5.

## 4. Discussion

### 4.1. Image-Guided Radiotherapy

Image-guided radiotherapy (IGRT) is associated with an improvement in biochemical tumor control among high-risk patients [36] and there are several techniques available for IGRT [37]. There is no universally accepted ‘Gold standard’ in IGRT. Previous work conducted to assess the accuracy of various IGRT techniques in the case of the prostate (including 3DUS and CBCT) revealed that inter-modality measurements for prostate displacement are essentially equivalent (comparable to within 3-4 mm) [29]. The prostate displacement probability distributions utilized in this study are in good agreement with the literature [38] and are representative of clinical reality. If this study were to be repeated according to a probability distribution obtained from an alternative prostate imaging modality, there is no reason to suspect that profoundly different results would be produced. 

### 4.2. Intra-Fraction Prostate Displacement

Intra-fraction motion was not simulated in this study. Analysis of intra-fraction motion of the prostate for a large dataset of patients revealed that on average the prostate was displaced >3 and >5 mm for 13.2% and 3.1% of the total treatment time per fraction, respectively [39]. The median values were 1.4% and 0.0%, the minimum and maximum range of values was reported to be 0.0%–98.7% and 0.0%–98.6%, respectively. However, intra-fraction motion is not well correlated with dosimetric impact and is small for the majority of cases [40]. These findings suggest that inclusion of intra-fraction motion, dependent on dose rate, would alter somewhat the results for stability but not robustness in this study.

### 4.3. Dosimetric Consequences of Geometrical Realignment

Previous studies have shown that geometrical realignment in the case of the prostate reasonably well retrieves the planned dose distribution for IMRT [33] and PSPT [41], i.e., there is no need for a dose recalculation after alignment. For IMRT [33] optimized with isotropic margins of 7 mm, the CTV ΔD_95_, defined as the difference in dose delivered before and after realignment to 95% of the target volume, was reported to be restored to 0% for isocenter realignments of ≤11 mm in all planes of motion. It is rational to expect similar values for VMAT.

For PSPT [41] optimized with margins of 5 mm in the axial and 8 mm in the anterior–posterior (AP) and superior–inferior (SI) planes of motion, the CTV V_78_, defined as the volume percentage receiving the prescribed dose of 78 Gy, was reported to be completely restored for isocenter realignments ≤10 mm in the AP and SI planes of motion. It is reasonable to assume that the same would be true for IMPT with appropriate margins [42]. To confirm this, we recalculated the dose for all P-EBRT simulations in this study. Thus demonstrating that geometrical realignment is a reasonable strategy in P-EBRT.

### 4.4. The Radiobiological Models

Radiobiological response is dependent upon physical factors such as total dose, fractional dose, LET (linear energy transfer), and biological factors such as radiosensitivity, hypoxia status, and RBE. The TCP model utilized here provided excellent correlation between predicted and reported 5-year clinical outcomes in prostate cancer patients treated by IMRT, VMAT, PSPT and IMPT. The NTCP model is based upon 3D-conformal radiotherapy escalation studies of early-stage prostate cancer [8]. It is possible that the altered low and intermediate dose distributions obtained with IMRT, VMAT, PSPT and IMPT may override the model to an extent that future data collection, analysis and modification of the volume-related model may be necessary, as well as a more sophisticated approach to RBE in the case of P-EBRT. A 2 Gy(E) fractionation scheme was used in this study in order to respect the original fit of the NTCP model.

### 4.5. Patient Sample Size

Increased patient sample size is always desirable from a statistical viewpoint. Twenty-five patients are on the lower end of what is appropriate to draw firm conclusions and is a limiting factor of this study. However, the patient sample size is similar to previous in silico published studies [15,43,44,45]. Additionally, Kolmogorov–Smirnov tests at the 1% confidence level revealed the TCP_plan_ and NTCP_plan_ data to be normally distributed for each modality; this supports the assumption that our patient cohort is representative of the overall population.

### 4.6. In Silico Trials

In silico trials enable prospective identification of patients who will likely benefit, in this instance through evaluation of technologies (both imaging and treatment). They can improve efficiency (patient throughput), efficacy (outcomes), and economy (cost-effectiveness) by providing streamlined, consistent, and knowledge-driven tools to support clinical decision making. However, such approaches are only as strong as the models/data upon which they are founded [46,47].

### 4.7. Prostate Immobilization and Implantable Rectal Spacers

With regard to endorectal balloons (ERB), intra-fraction prostate displacement is significantly reduced and inter-fraction prostate displacement is not [48]. Therefore, the results presented here for stability and robustness should be valid when ERB are utilized. However, the results for NTCP may alter when ERB are employed. No patients within this in silico trial were planned with ERB. Implantable rectal spacers (IRS) have been developed to temporarily create space between the rectal wall and the prostate during irradiation [49,50], thereby reducing the dose to the anterior rectum [51,52]. IRS would reduce the NTCP and probably alter the displacement characteristics of the prostate, potentially changing the findings of this study. No patients within this in silico trial were planned with IRS.

### 4.8. The Threshold for Likely Clinical Benefit

The results obtained at the cohort level reported in Table 2 enabled the determination of a threshold for likely clinical benefit, in this instance 5%. This value was calculated as 1.96 times the average difference between X-EBRT and P-EBRT across all possible scenarios, rounded down to the nearest integer. This threshold ensures that patients stratified into P-EBRT would have a predicted score of almost twice the average expected benefit at the cohort level, providing a level of confidence that patients stratified into P-EBRT will benefit from the treatment modality and strategy. However, this threshold can be arbitrarily lowered or raised in line with alternative rational, protocol or policy. Please see supplementary material: Tables S2–S7 and Figure 4.

### 4.9. Future Work

The results derived through the 25 patients analyzed in this study appear reasonable, representative and actionable. However, the results of this study should be replicated in similar or larger cohorts to mitigate the effect of unaccounted influencing factors, improving the statistics and reducing uncertainty, therefore increasing confidence in both the methodology and the results. Beyond that, to provide the highest possible level of evidence for the utility of such a clinical decision support system it should be validated in a prospective clinical trial. Additionally, application of the methodology presented here to the scenario of Carbon-ion therapy, as well as extending the modelling to incorporate cost-effectiveness, would be worthy contributions.

## 5. Conclusions

The results of this study predict that for these clinical conditions, planning criteria, radiobiological models, and simulations parameters: IMPT with image guidance is predicted to be the most effective treatment modality. Irrespective of the imaging system, each treatment modality is similar in terms of robustness, with and without the correction strategies. Conversely, there is substantial variation in stability between the treatment modalities, which is greatly reduced with the correction strategies. Correction strategies, preferably online-correction, for inter-fraction prostate displacement appear essential to maintain normal tissue sparing whilst ensuring that the target receives the prescribed dose. However, image guidance is not routinely employed at present in most P-EBRT centers. We recommend the application of appropriate image guidance correction strategies in PSPT and IMPT. Finally, in circumstances where IGRT is not feasible, plan stability and robustness coupled with prostate immobilization techniques must also be seriously considered. This in silico trial provides a methodology which can be utilized in a clinical decision support system (CDSS) to justify the selection of proton (P-EBRT) or photon (X-EBRT) external beam radiotherapy in prostate cancer patients, by way of an in silico trial.

## Figures and Tables

**Table 1:** Dose Volume Histogram criteria for plan acceptance.

DVH Parameter	Objective/Constraint
**PTV-D_median_** **PTV-D_100_** **PTV-D_95_** **PTV-D_2_**	≥78.0 Gy(E) ≥68.0 Gy(E) ≥74.1 Gy(E) ≤83.5 Gy(E)
**CTV-D_median_** **CTV-D_100_**	≥79.0 Gy(E) ≥70.0 Gy(E)
**Rectum/Sigmoid-colon-D_mean_** **Rectum/Sigmoid-colon-D_2_** **Rectum/Sigmoid-colon-V_70_** **Rectum/Sigmoid-colon-V_65_** **Rectum/Sigmoid-colon-V_60_** **Rectum/Sigmoid-colon-V_50_** **Rectum/Sigmoid-colon-V_40_**	≤52.0 Gy(E) ≤76.0 Gy(E) ≤30% ≤48% ≤59% ≤68% ≤84%
**Bladder-D_2_**	≤80.0 Gy(E)
**Small-intestine-D_2_**	≤70.0 Gy(E)
**Femoral-head-D_2_** **Femoral-head-V_50_**	≤60.0 Gy(E) ≤5%
**Sur_5.0-V_60_**	0%

CTV: clinical target volume, PTV: planning target volume, Sur_5.0: skin—(PTV expanded by 5 cm), created to avoid hotspots in the surroundings. D_median_: median dose; D_mean_: mean dose; D_100,95,2_: dose received by 100%, 95% and 2% of the volume. V_70,65,60,50,40_: volumes receiving 70, 65, 60, 50 and 40 Gy(E) respectively.

**Table 2:** Inter-modality evaluation and ranking at the cohort level.

Modalities and strategies				No-Correction				eNAL				Online			
	TCP_plan_	NTCP_plan_	COIN_PTV_	Robustness	Stability	Score	Rank	Robustness	Stability	Score	Rank	Robustness	Stability	Score	Rank
	(%)	(%)	(%)	(%)	(%)	(%)		(%)	(%)	(%)		(%)	(%)	(%)	
IMRT_3DUS_	53 ± 12 (19–70)	12 ± 3 (7–17)	63 ± 4 (55–73)	97 ± 3 (89–100)	71 ± 15 (37–91)	28 ± 10 (10–52)	2nd–3rd	97 ± 2 (93–99)	87 * ± 6 **(68–95)**	35 * ± 10 **(10–51)**	2nd	100 * ± 0 **(99–101)**	99 * ± 0 **(97–100)**	41 * ± 11 **(12–59)**	2nd
IMRT_CBCT_				99 * ± 2 **(94–100)**	78 ± 14 (37–92)	32 ± 10 (10–52)	2nd	99 * ± 1 **(97–101)**	93 * ± 3 **(86–97)**	38 * ± 10 **(11–55)**	2nd	100 * ± 0 **(99–101)**	100 * ± 0 **(99–101)**	40 * ± 11 **(12–57)**	2nd
VMAT_3DUS_	49 ± 13 (14–67)	12 ± 3 (6–19)	70 * ± 4 **(61–77)**	100 * ± 1 **(97–102)**	72 ± 13 (38–88)	27 ± 9 (4–40)	4th	100 * ± 1 **(98–99)**	88 * ± 6 **(69–95)**	34 ± 11 (5–47)	3rd	101 * ± 0 **(100–103)**	99 * ± 0 **(99–100)**	37 * ± 13 **(5–56)**	3rd
VMAT_CBCT_				100 * ± 1 **(99–102)**	74 ± 15 (33–93)	28 ± 10 (4–48)	3rd–4th	101 * ± 1 **(99–103)**	97 * ± 2 **(93–99)**	37 * ± 12 **(5–53)**	3rd	101 * ± 0 **(100–102)**	99 * ± 0 **(99–100)**	37 * ± 13 **(5–56)**	3rd
PSPT_3DUS_	47 ± 16 (16–67)	11 ± 3 (6–18)	52 * ± 6 **(42–65)**	99 * ± 1 **(97–101)**	79 * ± 9 **(60–90)**	28 ± 10 (5–49)	2nd–3rd	98 ± 1 (95–99)	90 * ± 4 **(83–97)**	33 ± 11 (6–54)	4th	99 * ± 1 **(98–100)**	98 * ± 1 **(97–99)**	36 * ± 12 **(6–58)**	4th
PSPT_CBCT_				99 * ± 1 **(97–100)**	80 * ± 6 **(68–90)**	28 ± 9 (5–44)	3rd–4th	98 * ± 1 **(97–99)**	91 * ± 3 **(85–96)**	33 ± 11 (6–54)	4th	99 * ± 1 **(98–100)**	98 * ± 1 **(97–99)**	35 * ± 12 **(6–57)**	4th
IMPT_3DUS_	56 ± 11 (30–70)	10 * ± 3 **(5–17)**	46 * ± 6 **(39–59)**	98 ± 1 (96–101)	73 ± 7 (54–82)	32 ± 8 (15–49)	1st	99 * ± 1 **(97–101)**	89 * ± 5 **(70–94)**	41 * ± 10 **(19–59)**	1st	100 * ± 0 **(100–101)**	99 * ± 0 **(99–100)**	46 * ± 10 **(21–65)**	1st
IMPT_CBCT_				99 * ± 1 **(98–101)**	75 ± 12 (42–89)	34 ± 9 (16–57)	1st	99 * ± 2 **(94–102)**	94 * ± 2 **(89–97)**	44 * ± 10 **(20–62)**	1st	101 * ± 1 **(99–102)**	99 * ± 0 **(98–100)**	47 * ± 10 **(22–65)**	1st

* Two-tailed paired. *t*-test: significant difference at the 5% level from the IMRT_3DUS_ (No-correction) dataset. Reported as the Mean ± StdDev (Range) for 25 patients.

## Supplementary Materials

The following are available online at http://www.mdpi.com/2072-6694/10/2/55/s1, Table S1: Inter-fraction prostate displacement metrics for 3DUS and CBCT, Table S2: Stratification into best treatment technique according to the CDSS, Table S3: Stratification into worst treatment technique according to the CDSS , Table S4: Difference between the best and worst treatment technique according to the CDSS, Table S5: Difference in score between the best P-EBRT and best X-EBRT treatment techniques according to the CDSS (CBCT: no-correction), Table S6: Difference in score between the best P-EBRT and best X-EBRT treatment techniques according to the CDSS (CBCT: eNAL-correction), Table S7: Difference in score between the best P-EBRT and best X-EBRT treatment techniques according to the CDSS (CBCT: online-correction).

## Abbreviations

The following abbreviations are used in this manuscript:

3DUS	three-dimensional ultrasound
CBCT	cone beam computed tomography
CDSS	clinical decision support system
COIN	conformity index
CT	computed tomography
CTV	clinical target volume
DVH	dose volume histogram
eNAL	extended no-action limit
ERB	endorectal balloon
Gy	Gray
Gy(E)	isoeffective Gray equivalent
ICRU	international commission on radiation units and measurements
IGRT	image-guided radiotherapy
IMPT	intensity-modulated proton therapy
IMRT	intensity-modulated radiotherapy
IRS	implantable rectal spacer
LET	linear energy transfer
MV	Megavolt
NTCP	normal tissue complication probability
OAR	organ at risk
P-EBRT	proton external beam radiotherapy
PSPT	passive scattered proton therapy
PTV	planning target volume
QUANTEC	quantitative analyses of clinical normal tissue effects criteria
RBE	relative biological effectiveness
ROCOCO	radiation oncology collaborative comparison
TCP	tumor control probability
TP	treatment planning
VMAT	volumetric arc therapy
X-EBRT	photon external beam radiotherapy
