# Supplementary material for: Towards a Clinical Decision Support System for External Beam Radiation Oncology Prostate Cancer Patients: Proton vs. Photon Radiotherapy? A Radiobiological Study of Robustness and Stability
Source: Cancers (Basel). 2018 Feb 18;10(2):55. doi: 10.3390/cancers10020055 (PMC5836087; doi:10.3390/cancers10020055)
Supplement: Supplementary file 1 [file cancers-10-00055-s001.pdf]

cancers

MDPI

Supplementary Materials: Towards a Clinical Decision Support System for External Beam Radiation Oncology Prostate Cancer Patients: Proton vs. Photon Radiotherapy? A Radiobiological Study of Robustness and Stability

Seán Walsh, Erik Roelofs, Peter Kuess, Yvonka van Wijk, Ben Vanneste, Andre Dekker, Philippe Lambin, Bleddyn Jones, Dietmar Georg and Frank Verhaegen

The document begins with a summary of the contouring protocol for the prostate utilized in this study, followed by the equation used to calculate the COIN metric. The prostate displacement statistics for both 3DUS and CBCT relative to initial skin-mark-laser alignment are given in **Table S1**. Additionally, **Tables S2–S3** shows the clustering of patients into their respective best and worst treatment modalities, while **Table S4** shows the difference in scores between these modalities. **Tables S5–S7** show the recommendations of the CDSS for each patient in the context of the correction strategies and displacement characteristics.

Contouring

Structure delineation was performed by consensus reading between a radiation oncologist and a radiologist, based upon T2 weighted MRI scans subsequently fused with CT datasets. The CTV constituted the prostate and seminal vesicles.

Conformity index

The conformity index (COIN) [28] provides a quantitative evaluation of the degree of conformity and was calculated for the PTV for each treatment plan for each modality.

$$COIN = \frac{V_{t_{dref}}}{V_t} \times \frac{V_{t_{dref}}}{V_{dref}} \quad (4)$$

Here, V_t is the volume of the target, d_{ref} is the reference dose level, V_{tdref} is the volume of the target receiving a dose ≥ d_{ref} and V_{dref} is the volume receiving a dose d ≥ d_{ref}. The first term of equation (4) represents the coverage of the target volume. The second term refers to the volume of healthy tissues receiving a dose d ≥ d_{ref}. COIN increases with conformity (0 ≤ COIN ≤ 1). It is important to note that COIN ≈ 0 when poor conformity is achieved (V_{dref} >> V_{tdref}) or when a geometrical miss occurs (V_{tdref} ≈ 0).

Prostate displacement relative to initial skin-mark-laser alignment

Table S1. Inter-fraction prostate displacement metrics for 3DUS and CBCT.

	−Left+Right (mm)	−Anterior+Posterior (mm)	−Superior+Inferior (mm)
M_{3DUS}	−0.8	−0.7	0.2
M_{CBCT}	1.1	−1.2	0.2
$\sum_{3DUS}$	±2.0	±3.5	±2.1
$\sum_{CBCT}$	±2.4	±3.0	±2.7
ξ_{3DUS}	±3.2	±3.7	±3.5
ξ_{CBCT}	±2.5	±3.2	±2.2

M: The group mean displacement; ∑: The systematic error component is the standard deviation of all patients' mean displacement values; ξ: The random error component is the root measn square of all patients' standard deviation displacement values.

Patient stratification

Table S2. Stratification into best treatment technique according to the CDSS.

Correction	IMRT	VMAT	PSPT	IMPT	Total
No-Correction$_{CBCT/3DUS}$	4/4	2/2	2/3	17/16	25/25
eNAL$_{CBCT/3DUS}$	0/0	4/3	0/1	21/21	25/25
Online$_{CBCT/3DUS}$	2/2	1/1	0/0	22/22	25/25

Table S3. Stratification into worst treatment technique according to the CDSS.

Correction	IMRT	VMAT	PSPT	IMPT	Total
No-Correction$_{CBCT/3DUS}$	1/5	12/10	9/8	3/2	25/25
eNAL$_{CBCT/3DUS}$	1/4	6/9	18/12	0/0	25/25
Online$_{CBCT/3DUS}$	2/1	9/9	14/15	0/0	25/25

Table S4. Difference between the best and worst treatment technique according to the CDSS.

Correction	Diff ≥ 5%	Diff ≥ 10%	Diff ≥ 20%
No-Correction$_{CBCT/3DUS}$	23/23	11/14	3/1
eNAL$_{CBCT/3DUS}$	23/24	12/14	0/1
Online$_{CBCT/3DUS}$	25/25	15/15	0/0

Table S5. Difference in score between the best P-EBRT and best X-EBRT treatment techniques according to the CDSS (CBCT: no-correction).

Patient	Best Modality	Magnitude Difference
1	'PSPT'	17.16
2	'IMRT'	1.54
3	'IMRT'	−4.19
4	'IMRT'	3.16
5	'PSPT'	7.12
6	'IMPT'	5.90
7	'VMAT'	3.35
8	'IMRT'	2.34
9	'IMPT'	5.41
10	'VMAT'	−14.88
11	'IMRT'	2.13
12	'IMRT'	0.31
13	'IMRT'	−8.01
14	'IMPT'	5.04
15	'VMAT'	−0.46
16	'VMAT'	2.21
17	'IMRT'	1.79
18	'IMPT'	6.11
19	'IMRT'	−1.04
20	'IMRT'	4.82
21	'IMRT'	3.38
22	'IMRT'	0.60
23	'IMRT'	4.00
24	'IMRT'	−1.92
25	'IMRT'	3.00

Table S6. Difference in score between the best P-EBRT and best X-EBRT treatment techniques according to the CDSS (CBCT: eNAL-correction).

Patient	Best Modality	Magnitude Difference
1	'VMAT'	−1.12
2	'IMRT'	0.88

3	'VMAT'	−0.11
4	'IMRT'	2.81
5	'VMAT'	−2.20
6	'IMPT'	6.62
7	'VMAT'	3.63
8	'IMPT'	6.89
9	'IMRT'	3.85
10	'VMAT'	−1.57
11	'IMPT'	5.54
12	'IMRT'	1.16
13	'IMPT'	5.03
14	'IMPT'	7.14
15	'IMRT'	2.46
16	'VMAT'	3.94
17	'IMRT'	2.89
18	'IMPT'	9.16
19	'IMRT'	4.10
20	'IMPT'	6.76
21	'IMRT'	3.13
22	'IMRT'	3.17
23	'IMPT'	5.21
24	'IMRT'	3.36
25	'IMRT'	3.59

Table S7. Difference in score between the best P-EBRT and best X-EBRT treatment techniques according to the CDSS (CBCT: online-correction).

Patient	Best Modality	Magnitude Difference
1	'IMRT'	−1.18
2	'IMRT'	−1.16
3	'VMAT'	0.85
4	'IMRT'	4.37
5	'VMAT'	−0.55
6	'IMPT'	7.05
7	'IMPT'	5.89
8	'IMPT'	8.07
9	'IMRT'	3.06
10	'VMAT'	0.80
11	'IMPT'	6.01
12	'IMRT'	1.16
13	'IMPT'	5.46
14	'IMPT'	7.12
15	'IMRT'	2.81
16	'VMAT'	4.72
17	'IMRT'	4.68
18	'IMPT'	9.96
19	'IMPT'	5.85
20	'IMPT'	8.28
21	'IMRT'	2.81
22	'IMRT'	0.60
23	'IMRT'	4.00
24	'IMRT'	−1.92
25	'IMRT'	3.00

© 2018 by the authors. Licensee MDPI, Basel, Switzerland. This article is an open access article distributed under the terms and conditions of the Creative Commons Attribution (CC BY) license (http://creativecommons.org/licenses/by/4.0/).
